# 56 Prevalence and severity of infections in patients with systemic autoimmune diseases on biotherapy

**DOI:** 10.1093/rheumatology/keac496.052

**Published:** 2022-10-07

**Authors:** Ben Ayed Hiba, Ben Yacoub Sarra, Boudokhane Manel, Bourguiba Rym, Teyeb Zeineb, Ayari Mariem, Jomni Taieb, Abdelaali Imen, Belakhal Syrine, Dougui Mohamed Hedi

## Abstract

**Introduction:**

The prognosis of chronic inflammatory diseases has been transformed by the use of biologic drugs. However, there is evidence of an increased risk of infection in patients undergoing biotherapy. The objective of our study was to investigate the frequency and the severity of infections in patients on biotherapy.

**Methods:**

We conducted a retrospective descriptive study including all patients followed in the internal medicine department of the ISF hospital and treated with biotherapy over a period of 14 years [2009–2022].

**Results:**

We identified 50 patients treated with biologic therapy among 76 patients. The patient’s mean age was 48 years± 12.5 [23–76] and male to female ratio (M: F) was 1 :6. The mean age at initiation of biotherapy was 41.3 ± 11.6 [17–68] years. The molecules used were TNFα inhibitors in 82% of cases, rituximab in 14% of cases and tocilizumab in 4%. Infectious complications were identified in 20 patients. Infections occurred after a mean duration of 12 months [0.5–36] after the initiation of biotherapy. They affected the skin in nine cases, the bronchopulmonary tract in seven cases, the central nervous system in four cases, the gastrointestinal tract in three cases, and the upper respiratory tract in two cases. The infection was bacterial in 16 cases (64%), viral in five cases (20%) and fungal in four cases (16%). We report five cases of tuberculosis (TB), including four cases of pulmonary TB and one case of neuro-meningeal TB. The infection was severe, requiring hospitalization in only eight cases (32%). The median duration of hospitalization was 12 days [2–58]. The infectious episode led to temporary interruption of biotherapy in six cases and definitive interruption in two cases. No deaths were noted.

**Conclusion:**

Our study confirms the increased susceptibility to infections in patients treated with biologics. These infections, often bacterial, are in the majority of cases benign. However, tuberculosis remains frequent.

